# Overdiagnosis and overtreatment of thyroid cancer: A population-based temporal trend study

**DOI:** 10.1371/journal.pone.0179387

**Published:** 2017-06-14

**Authors:** Sabrina Jegerlehner, Jean-Luc Bulliard, Drahomir Aujesky, Nicolas Rodondi, Simon Germann, Isabelle Konzelmann, Arnaud Chiolero, NICER Working Group

**Affiliations:** 1 Division of General Internal Medicine, Inselspital, Bern University Hospital, University of Bern, Bern, Switzerland; 2 Division of Chronic Diseases, IUMSP, Lausanne University Hospital, Lausanne, Switzerland; 3 Institute of Primary Health Care (BIHAM), University of Bern, Bern, Switzerland; 4 Observatoire valaisan de la santé (OVS), Sion, Switzerland; 5 Department of Epidemiology, McGill University, Montreal, Canada; Tel Aviv Sourasky Medical Center, ISRAEL

## Abstract

**Background:**

The increase in incidence of thyroid cancer during the last decades without concomitant rise in mortality may reflect the growing detection of indolent forms of thyroid cancer, and may have fueled unnecessary thyroidectomies. Our aim was therefore, to compare recent secular trends in surgical intervention rate for thyroid cancer with the incidence and mortality of thyroid cancer to assess overdiagnosis and resulting overtreatment.

**Methods:**

We conducted a population-based temporal trend study in Switzerland from 1998 to 2012. All cases of invasive thyroid cancer, deaths from thyroid cancer, and cancer-related thyroidectomies were analyzed. We calculated changes in age-standardized thyroid cancer incidence rates, stratified by histologic subtype and tumor stage, thyroid cancer-specific mortality, and thyroidectomy rates.

**Results:**

Between 1998 and 2012, the age-standardized annual incidence of thyroid cancer increased from 5.9 to 11.7 cases/100,000 among women (annual mean absolute increase: +0.43/100,000/year) and from 2.7 to 3.9 cases/100,000 among men (+0.11/100,000/year). The increase was limited to the papillary subtype, the most indolent form of thyroid cancer. The incidence of early stages increased sharply, the incidence of advanced stages increased marginally, and the mortality from thyroid cancer decreased slightly. There was a three- to four-fold increase in the age-standardized annual thyroidectomy rate in both sexes.

**Conclusions:**

We observed a large increase in the incidence of thyroid cancer, limited to papillary and early stage tumors, with a three- to four-fold parallel increase in thyroidectomy. The mortality slightly decreased. These findings suggest that a substantial and growing part of the detected thyroid cancers are overdiagnosed and overtreated.

**Impact:**

Targeted screening and diagnostic strategies are warranted to avoid overdetection and unnecessary treatment of thyroid cancers.

## Introduction

With an estimated 316,000 new cases in 2015, thyroid cancer represents about 2% of all invasive cancers with a large between-country variability [1]. In North America, the age-standardized incidence rate of thyroid cancer was 20.0 cases per 100,000 among women and 6.3 cases per 100,000 among men in 2012 [1]. Thyroid cancer has an excellent vital prognosis with a 5-year survival rate of above 85% [2, 3]. Over the last few decades, the incidence of thyroid cancer has markedly increased in high-income countries [4], including the United States [5], South Korea [6], the United Kingdom and several European countries [7–11]. It is the cancer with the fastest increase in frequency and if recent trends are maintained, thyroid cancer will be the fourth most common cancer by 2030 [12]. Further, in several studies, the increase in incidence was limited to papillary carcinoma, the most frequent and indolent histological form of thyroid cancer [10, 13], and the thyroid cancer-specific mortality rate has remained stable or slightly decreased [4–16]. An increase in incidence largely confined to the more indolent histological subtype and to early tumour stages, without concomitant increase in mortality, suggests cancer overdiagnosis, that is the detection of indolent cancer forms that will neither cause symptoms during a persons’ lifetime, nor reduce lifespan [13–18].

Because most thyroid cancers are primarily treated by surgery, i.e., partial or total thyroidectomy [19], overdiagnosis leads to overtreatment through the performance of unnecessary thyroidectomies without clear benefit for the patient. Incidence and mortality based studies supporting overdiagnosis [4–18] on the one side, and hospital-based series of increased number of thyroidectomies performed [20–22] on the other side, have been reported. However, no study has assessed concomitant trends in the incidence and mortality of thyroid cancer and related surgery at a population level.

Our aim was therefore to compare recent secular trends in surgical intervention rate for thyroid cancer with the incidence and mortality of invasive thyroid cancer in Switzerland. Our hypothesis was that an increase in the incidence of papillary subtype and early-stage thyroid tumors with a concomitant increase in thyroidectomy rates, but without increase in thyroid cancer-specific mortality, would provide indirect evidence for thyroid cancer overdiagnosis and overtreatment.

## Materials and methods

### Study design

We conducted population-based temporal trend analyses of incidence and mortality rates of thyroid cancer and of thyroidectomy rates in Switzerland between 1998 and 2012.

### Data sources and case definition

#### 1. Registry data for cancer cases and mortality

In Switzerland, registration of cancer is organized at the regional level with a high level of exhaustivity. Data on all new cancer cases are collected, documented and recorded by population-based regional cancer registries. The National Institute for Cancer Epidemiology and Registration (NICER) compiles and aggregates this data (www.nicer.org). Quality control procedures are based on the guidelines from the European Network of Cancer Registries [23]. In 2012, regional cancer registries covered 68% of the Swiss population and all contributed to this study (cantons Appenzell (Inner and Ausser), Basel (Stadt and Land), Geneva, Glarus, Graubünden, Jura, Luzern, Neuchâtel, Obwalden and Nidwalden, St. Gallen, Thurgau, Ticino, Uri, Vaud, Valais, Zug and Zürich). Cancer mortality data based on death reports are collected and checked by the Swiss Federal Statistical Office (SFSO), and were provided by NICER for this study.

Neoplastic pathologies are coded according to the 3^rd^ edition of the International Classification of Diseases for Oncology (ICD-O-3). For our analyses, we used primary invasive thyroid cancer cases (ICD-O-3: C73) recorded in all cancer registries between 1998 and 2012. Thyroid cancers were grouped by histological subtype (papillary: ICD-O-3 codes 8050, 8260, 8340, 8341, 8342, 8343, 8344 and 8350; non-papillary: ICD-O-3 codes 8020, 8021, 8030, 8031, 8032, 8033, 8041, 8290, 8330, 8331, 8332, 8335, 8345, 8346, 8347, 8510, 8511 and 8512; rare (<1%) and unknown types: others/unknown) and tumor stage (early, advanced) according to recent oncology guidelines using the TNM classification (European Society for Medical Oncology (ESMO; www.esmo.org), National Comprehensive Cancer Network (NCCN; www.nccn.org), American Thyroid Association (ATA) [19], TNM (www.uicc.org/resources/tnm)). In persons younger than 45 years of age, stage I (M0) tumors were defined as early cancer and stage II (M1) tumors were defined as advanced cancer. In persons aged 45 or older, stage I (T1N0M0) and stage II tumors (T2N0M0) were defined as early cancer whereas stage III (T3N0M0, T1-3N1aM0) and stage IV (M1, all anaplastic carcinoma) tumors were defined as advanced cancer [19].

Trends by stage were limited to Swiss regions for which staging was documented in over 90% of cases each year (Basel-Stadt, Basel-Land, Fribourg, Geneva and Valais) to allow reliable analyses. By focusing on invasive thyroid cancer cases diagnosed between 1998 and 2012, our analyses were not affected by the recent histological reclassification of encapsulated follicular variant of papillary cancer to noninvasive follicular neoplasm with papillary-like nuclear features [24].

#### 2. Hospital data for thyroid cancer surgery

Data on thyroid cancer surgery were collected from all Swiss inpatient cases using the SFSO’s Hospital Medical Statistics. Surgical procedures are registered by a year-specific Swiss Classification of Surgical Interventions (CHOP) code [25]. Codes are determined by physicians and checked by trained medico-administrative staff to ensure their accuracy and completeness. In 2012, 99% of all hospitals in Switzerland were included with a case coverage of 98% of all admissions.

To analyze trends in surgery between 1998 and 2012, we used year-specific diagnostic ICD-O-3 and CHOP codes. We identified individuals with a diagnosis of invasive thyroid cancer (ICD-O-3 code: C73) who had major cancer-related surgery, that is, partial or total thyroidectomy (CHOP code: 06.2–06.5; unilateral hemithyroidectomy, other partial thyroidectomy, total thyroidectomy, substernal thyroidectomy) during the same year.

#### Assessment of overdiagnosis

There is no direct way to prove that a cancer has been overdiagnosed unless people are followed without treatment until they die of other causes. Secular trends incidence and mortality rates can however provide indirect evidence for overdiagnosis [15]. A substantial and sustained increase in incidence without concomitant increase in mortality of, and accordingly a parallel increase in surgery for, thyroid cancer are indirect evidence of overdiagnosis and resulting overtreatment, respectively. A substantial and sustained increase in incidence of early stage cancer without an increase in the incidence of advanced stage cancer can also be suggestive of overdiagnosis.

#### Statistical analyses

Rates were age-standardized to the European standard population. We computed annual thyroid cancer incidence rates stratified by sex, histological subtype, and tumour stage. We further computed annual thyroid cancer mortality rates and thyroidectomy (partial or total) rates stratified by sex. To smooth estimates, we computed absolute differences in using pooled data from the first three years (1998–2000) and the last three years (2010–2012). We fitted a linear regression model to estimate the annual mean absolute and relative changes in the standardized rates with calendar year as predictor variable. Joinpoint statistical software (version 4.3; Surveillance Research Program, National Cancer Institute, Bethesda, MD) was used to identify and estimate the parameters of the linear model and to test for statistical significance. In Joinpoint, we used the grid search method and provided standard errors to adjust for heterosedascticity. To account for the possibility of incomplete recording in the three first years of the study period, a set of sensitivity analyses were conducted omitting data collected between 1998 and 2000. Statistical analyses were performed with STATA (version 13) and R-studio.

#### Ethics

Anonymised and publicly available aggregated data were used for our analyses. There was no threat to patient confidentiality. According to the Swiss Human Research Act (Humanforschungsgesetz HFG), no ethical approval or trial registration is needed for such analyses. Our study protocol was agreed upon by NICER. The SFSO allowed the analyses and publication of deidentified hospital data (contract 150 556).

## Results

**Fig 1:**
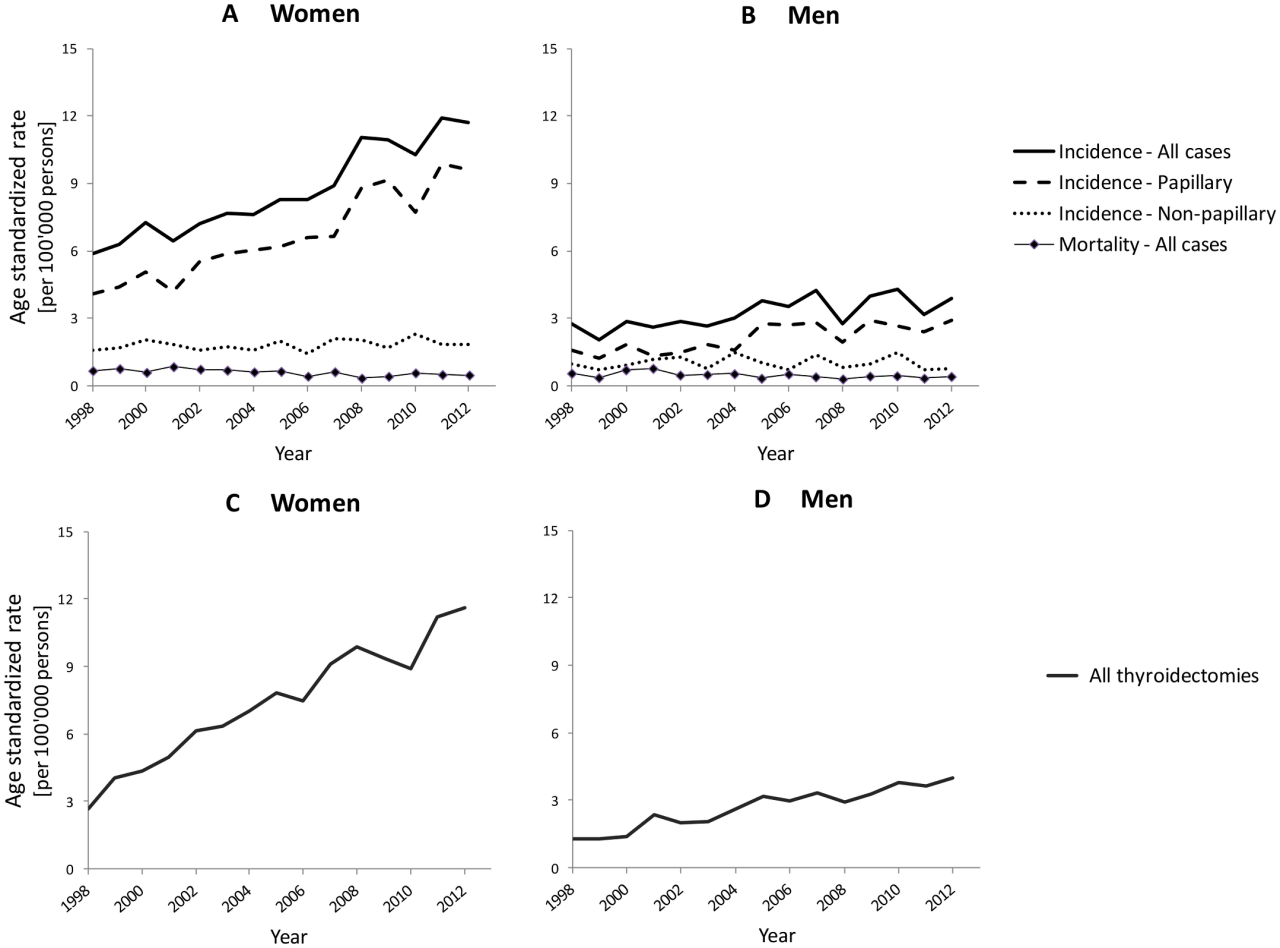
Age-standardized incidence and age-standardized mortality rates for thyroid cancer in women (A) and men (B). Age-standardized rate of thyroidectomy in women (C) and men (D), Switzerland, 1998–2012. Data source: National Institute of Cancer Epidemiology and Registration (NICER) and Swiss Federal Statistical Office (SFSO) Hospital Medical Statistics.

Between 1998 and 2012, 4,907 cases of thyroid cancer (women: 3,642; men: 1,265) were registered and analyzed. The total number of cases by year, sex, and histological subtype are reported in S1 Table. The age-standardized incidence rate of thyroid cancer was about three times higher in women than in men (Fig 1 **panel A and B**; Tables 1 and 2). Between 1998 and 2012, it increased by 7% per year in women, corresponding to an absolute mean annual change of +0.43/100,000 women (95% CI: 0.37 to 0.49) (Table 3). During the same time period, the incidence of thyroid cancer increased by 4% per year in men, corresponding to an absolute mean annual change of +0.11/100,000 men (95% CI: 0.07 to 0.15).

**Table 1:** Age-standardized (European population) incidence rates, mortality rates, and thyroidectomies for thyroid cancer per 100’000 inhabitants, by year and sex. Switzerland, 1998–2012. Incidences are also reported by histological subtypes.

Year	Women					Men				
	Incidence			Mortality	Thyroidectomies	Incidence			Mortality	Thyroidectomies
	Papillary	Non-papillary	All cases	All cases	All cases	Papillary	Non-papillary	All cases	All cases	All cases
1998	4.07	1.79	5.86	0.67	2.68	1.59	1.15	2.74	0.56	1.27
1999	4.40	1.90	6.30	0.76	4.06	1.21	0.80	2.02	0.36	1.29
2000	5.06	2.18	7.24	0.59	4.34	1.82	1.05	2.88	0.71	1.39
2001	4.18	2.24	6.42	0.85	4.98	1.31	1.27	2.58	0.78	2.33
2002	5.51	1.72	7.23	0.74	6.11	1.50	1.34	2.84	0.48	2.00
2003	5.89	1.80	7.69	0.69	6.36	1.90	0.80	2.70	0.50	2.06
2004	6.03	1.59	7.62	0.61	6.99	1.57	1.47	3.04	0.53	2.61
2005	6.16	2.10	8.26	0.64	7.84	2.74	1.05	3.80	0.33	3.19
2006	6.61	1.66	8.27	0.43	7.44	2.71	0.84	3.55	0.52	2.94
2007	6.64	2.24	8.88	0.62	9.12	2.80	1.43	4.24	0.40	3.30
2008	8.81	2.23	11.04	0.34	9.86	1.92	0.85	2.77	0.31	2.89
2009	9.14	1.79	10.93	0.42	9.33	2.93	1.05	3.97	0.43	3.26
2010	7.74	2.55	10.29	0.57	8.91	2.67	1.62	4.29	0.44	3.79
2011	9.88	2.04	11.93	0.50	11.21	2.38	0.78	3.16	0.34	3.63
2012	9.61	2.10	11.71	0.48	11.61	2.93	0.95	3.88	0.42	4.00
**Absolute change 1998–2000 vs 2010–2012**	+4.6	+0.2	+4.9	-0.2	+6.88	+1.1	+0.1	+1.2	-0.2	+2.49
**Relative change 1998–2000 vs 2010–2012**	+102%	+13%	+75%	-24%	+186%	+73%	+13%	+48%	-27%	+189%

Data source: National Institute of Cancer Epidemiology and Registration (NICER) and Swiss Federal Statistical Office (SFSO), Hospital Medical Statistics.

**Table 2:** Age-standardized (European population) incidence rates for thyroid cancer, per 100’000 inhabitants, by year, sex and stage, Switzerland, 1998–2012.

Year	Women Incidence		Men Incidence	
	Early	Advanced	Early	Advanced
1998	6.41	1.49	1.30	0.97
1999	6.58	1.26	1.29	0.65
2000	5.50	1.85	1.80	1.07
2001	5.06	1.91	1.19	0.65
2002	5.76	1.20	1.83	0.61
2003	6.86	1.96	2.30	0.93
2004	7.22	1.21	1.90	1.57
2005	9.01	1.28	3.66	1.27
2006	6.29	1.26	3.67	0.95
2007	7.69	1.16	3.66	1.98
2008	9.76	2.16	2.63	1.26
2009	11.18	2.47	2.69	2.15
2010	9.90	1.39	3.16	1.55
2011	12.15	1.28	1.73	0.99
2012	12.46	2.44	2.86	1.12
**Absolute change 1998–2000 vs 2010–2012**	+ 5.34	+ 0.18	+ 1.12	+ 0.33
**Relative change 1998–2000 vs 2010–2012**	+ 87%	+ 12%	+ 76%	+ 37%

Data source: National Institute of Cancer Epidemiology and Registration (NICER), limited to 5 regions (cf. Methods section).

**Table 3:** Trends in the incidence and mortality of thyroid cancer and in the incidence of thyroidectomy, 1998–2012; Switzerland. All rates are directly standardized on the European population. CI: confidence interval.

	Women				Men			
	Absolute annual mean change in rate [per 100’000]	95% CI [per 100’000]	Relative increase per year	95% CI [%]	Absolute annual mean change in rate [per 100’000]	95% CI [per 100’000]	Relative increase per year	95% CI [%]
**Incidence**	+ 0.43	0.37 to 0.49	+ 7%	6% to 8%	+ 0.11	0.07 to 0.15	+ 4%	3% to 6%
**Incidence by histologic type**_a_								
Papillary	+ 0.41	0.35 to 0.47	+ 10%	9% to 12%	+ 0.11	0.08 to 0.15	+ 8%	6% to 11%
Non-papillary	+ 0.02	0.00 to 0.04	+ 1%	0% to 2%	0.00	-0.03 to 0.02	- 1%	- 2% to 2%
Other/unknown	+ 0.01	-0.01 to 0.02	0%	- 2% to 7%	+ 0.01	0.00 to 0.02	+ 17%	- 9% to 44%
**Incidence by stage**_b_								
Early	+ 0.47	0.32 to 0.62	+ 9%	6% to 12%	+ 0.11	0.04 to 0.19	+ 7%	3% to 13%
Advanced	+ 0.02	-0.03 to 0.06	+ 1%	- 2 to 4%	+ 0.05	0.02 to 0.09	+ 6%	2% to 11%
Unknown	0.00	0.00 to 0.01	0%	-	0.00	0.00 to 0.00	0%	-
**Mortality**_a_	- 0.02	-0.03 to -0.01	- 3%	- 4% to - 2%	- 0.01	-0.02 to 0.00	- 2%	- 4% to - 2%
**Surgery incidence**_c_								
Thyroidectomy	+ 0.59	0.53 to 0.64	+ 17%	16% to 19%	+ 0.20	0.17 to 0.23	+ 15%	13% to 17%

Data sources:

^a^National Institute of Cancer Epidemiology and Registration (NICER);

^b^NICER limited to 5 regions (cf. Methods section);

^c^Swiss Federal Statistical Office (SFSO), Hospital Medical Statistic

The increase in incidence was essentially due to an increase in the papillary subtype (Fig 1 **Panel A, B**; Table 1). Between 1998 and 2012, the age-standardized incidence of papillary carcinoma increased by 10% per year in women, corresponding to an annual mean change of +0.41/100,000 women (95% CI: 0.35 to 0.47). In men, the incidence increased by 8% per year, corresponding to an annual mean change of +0.11/100,000 men (95% CI: 0.08 to 0.15). In both sexes, there was no substantial change in the incidence of other histologic subtypes of thyroid cancer (Table 3).

The absolute increase in the incidence of early stages of thyroid cancer was large, especially in women (Tables 2 and 3). No statistically significant increase was observed for advanced stages in women, while a small absolute increase was observed in men.

Between 1998 and 2012, 592 deaths from thyroid cancer (women 397; men: 195) occurred. In contrast to the rising incidence, the thyroid cancer-related mortality slightly decreased in both sexes (Fig 1 **Panel A, B**; and Table 1). In women, the age-standardized mortality rate decreased by 3% per year, corresponding to an annual mean absolute change of -0.02/100,000 women (95% CI: -0.03 to -0.01). In men, the mortality rate decreased by 2% per year, corresponding to annual mean absolute change of -0.01/100,000 men (95% CI: -0.02 to 0.00).

Between 1998 and 2012, 6,419 cases of thyroidectomy (women: 4,763; men: 1,656) were registered in Switzerland (S2 Table). In both sexes, a large increase in the rate of thyroidectomy was observed (Fig 1 **Panel C, D**; and Table 1). The age-standardized rate of thyroidectomy increased yearly by 17% in women, corresponding to an annual mean absolute change of +0.59/100,000 women (95% CI: 0.53 to 0.64), and by 15% in men, corresponding to an annual mean absolute change of +0.20/100,000 men (95% CI: 0.17 to 0.23) (Table 3).

## Discussion

Our study demonstrates a large rise in the incidence of thyroid cancer, confined to papillary carcinoma and early stage tumors, with a concomitant three- to four-fold increase in the rate of thyroidectomy and a slight decrease in thyroid cancer mortality in Switzerland between 1998 and 2012. These findings suggest that a substantial and growing part of the detected thyroid cancers are overdiagnosed and overtreated.

Our results are consistent with several studies showing a rapidly increasing thyroid cancer incidence without a substantial change or a slight decrease in mortality in high-income countries [4, 7–11, 16, 26, 27]. In recent analyses comparing expected versus observed incidence, 45% to 70% of thyroid cancer cases were considered as overdiagnosed in France, South Korea, the United Kingdom and the United States [28]. The magnitude of our observed increase in the incidence of thyroid cancer over the last 15 years in Switzerland is close to the reported increase in the United States, but far less than in South Korea, where thyroid cancer screening with ultrasonography as health checkup is commonly performed by general practitioners since 1999 [6]. Trend analyses of thyroid cancer surgery are however much rarer [20–22, 29]. In the United States, hospital data revealed a 31% increase in the number of total thyroidectomies or lobectomies performed for thyroid nodule-related diagnoses between 2006 and 2011 [20]. Another report using discharge data from the Nationwide Inpatient Sample showed a 39% increase in the number of thyroid surgical cases for benign and malignant diseases between 1993 and 2008 with a growing proportion of operations performed for cancer [29]. In Illinois, the rate of thyroidectomies increased by 44% from 1999 to 2009 [21]. However, these rates were not age-standardized. A study from Belgium showed a correlation between thyroid surgery of any indication (mainly goiters) and detection of low-risk thyroid cancer after histologic work-up [30]. To our knowledge, our study is the first to report nationwide, population-based, and age-standardized estimates of the incidence of thyroid cancer and concomitant related surgery.

We cannot exclude that unfavorable trends in risk factors for thyroid cancer could explain our observations. Radioactive exposure is a major risk factor, particular for papillary thyroid cancer [31]. Despite an increase in exposure from medical conditions, the overall radiation burden in Switzerland has declined following broad surveillance and protection measures [32]. The impacts on thyroid cancer rate of the nuclear power plant accident of Chernobyl in 1986 are difficult to assess. After this disaster, an increase in thyroid cancer was reported among children and adolescents exposed to radioactive iodine in Belarus, Russia, and the Ukraine [33]. In Switzerland, it was estimated that the impact of this accident on cancer in Switzerland was rather low, with an estimated 200 cases of additional cancer deaths of all types and with unknown effect on thyroid cancers [34]. Such an effect could not explain the large increase in the number of thyroid cancers observed in our study. Shortly after the Fukushima Daiichi nuclear plant accident in 2011, screening with thyroid ultrasound examination was implemented [35]. There was a large increase in the detection of thyroid cancer. However, this increase has been attributed mainly to overdiagnosis caused by the screening itself, as the time lag between the exposure and the increase in the incidence was very short, and as there was no direct correlation between the increase in the incidence and the regional level of radiation exposure [36].

Another risk factor for thyroid cancer is iodine deficiency, predisposing to goiter and formation of thyroid nodules. In Switzerland, the salt is iodized since 1922 to ensure sufficient supply. Obesity and other factors related to diet and environment, hormones and genetic background are inconsistently reported risk factors for thyroid cancer and unlikely to influence materially our results, but cannot be excluded. However, it would be difficult to explain how a change in any of these risk factors would increase only the frequency of early stage cases or indolent forms of thyroid cancer without increasing the incidence of advanced stage cases and the mortality rate. Changes in histopathologic diagnostic criteria for thyroid cancer in 1988 led to a spurious upward trend in incidence in one Swiss region, but seemed unlikely to affect our study period [32, 37].

The most likely cause of the rise in the incidence of thyroid cancer is increasing detection due to incidental findings through advanced imaging and the systematic diagnostic exploration of small thyroid nodules. Autopsy studies have shown that there is a large reservoir of subclinical papillary thyroid cancers [38]. Three mechanisms have been identified to explain the growing number of thyroid cancer diagnosed: opportunistic screening (by physical examinations of the thyroid in asymptomatic patients), diagnostic cascade (through performance of multiple tests to evaluate vague complaints) and incidental findings (through detection of thyroid abnormalities on radiological examinations not performed to examine the thyroid) [5]. Although there are no recommendations to screen systematically for thyroid cancer in Switzerland, ultrasonography and guided fine-needle biopsy in patients with thyroid nodules are commonly performed. The high availability of advanced imaging (computed tomography (CT) scanners and Magnetic Resonance Imaging (MRI) units) per inhabitant in Switzerland (www.oecd.org) facilitates the prescription, e.g., of neck MRI or chest CT with the resulting risk of incidental findings of thyroid nodules and, eventually, of thyroid cancer [38]. Had thyroid cancer incidence remained at the level of 1998 in Switzerland and assuming that all the increase in the incidence was due to overdiagnosed cases, then at least one third of thyroidectomies could be unnecessarily performed each year in Switzerland.

The slight decrease in thyroid cancer-specific mortality over time may reflect improved treatment strategies, thanks e.g. to refined surgical techniques with a lower complication rate and better post-surgical care. In contrast to our observations, Lim et al. recently described an increase in the overall incidence of thyroid cancer and related mortality in the US, such findings being consistent with a true (not only due to overdiagnosis) increase in thyroid cancer [39]. However, the absolute increase in mortality was very small compared with the huge increase in incidence, which points to a large contribution of overdiagnosis to the increasing incidence, as acknowledged by the authors. The increase in mortality could also indicate a worsening in quality of care and outcome of thyroid cancer surgery.

Our study has several strengths. First, our analyses rely on population-based and high quality data, including cancer registries and hospital-based statistics covering a large proportion of the population. Second, we analyzed trends in thyroid cancer and surgery for thyroid cancer during the same time period to compare incidence, mortality and surgery rates at a whole population level. However, our study has several limitations. As a time trend analysis, it can provide only indirect evidence of overdiagnosis and overtreatment. There is no direct way to prove that a cancer is overdiagnosed unless patients are followed untreated until they die of other causes [15–18]. Although the Swiss population coverage by cancer registries was not nationwide, to the best of our knowledge, there is no reason to believe that thyroid cancer trends differ substantially across regions in particular by histological type or stage. Thus, the restriction of tumor stage analyses to regions for which staging was nearly systematically documented (over 90% of cases each year) appears unlikely to alter substantially our results. Also, we cannot exclude that surgery coding practices may differ between hospitals and change across time. These are general and well-known issues of studies using medico-administrative data [40]. To minimize this risk, we have taken into account changes of coding procedures for thyroid surgery over time using year-specific codes. Further, as thyroid surgeries are major procedures performed as inpatient intervention, the probability of cases not being registered and coded is very low.

In conclusion, our study provides indirect evidence of overdiagnosis and overtreatment of thyroid cancer. It appears that a substantial number of thyroid cancers that were never destined to cause symptoms in a person’s life are detected and treated with potentially unnecessary surgery and considerable long-term consequences and risk of complications. Examination of the mechanisms that drive overdiagnosis and overtreatment of thyroid cancer is urgently warranted. Due notably to the harms of overdiagnosis, the US Preventive Services Task Force has recently updated its recommendation against routine screening for thyroid cancer (D level recommendation) [38, 41]. To identify which patients could benefit from the detection of early stage cases, a risk-based screening with combined diagnostic and prognostic tools (biomarker, personalized medicine) could be a promising approach to tailor prevention and screening according to the individual risk of thyroid cancer [42]. Further, active surveillance of incidental, asymptomatic and small papillary thyroid cancers should be seriously considered and tested in adequately designed clinical studies [7]. Meanwhile, patients should be informed of the overall excellent prognosis of the great majority of small papillary thyroid cancers, as well as of the uncertainties in our ability to identify “high risk” cancers [43].

## Supporting information

S1 Table Number of thyroid cancer cases by year, sex and histological subtype, Switzerland, 1998–2012. (DOCX)

S2 Table Number of thyroidectomy cases by year and sex, Switzerland, 1998–2012. (DOCX)
