# Supplementary material for: Overdiagnosis and overtreatment of thyroid cancer: A population-based temporal trend study
Source: PLoS One. 2017 Jun 14;12(6):e0179387. doi: 10.1371/journal.pone.0179387 (PMC5470703; doi:10.1371/journal.pone.0179387)
Supplement: S1 Table — (DOCX) [file pone.0179387.s001.docx]

**Supporting information**

**S1 Table.** Number of thyroid cancer cases by year, sex and histological subtype, Switzerland, 1998-2012.

| **Year** | **Papillary** | | **Non-papillary** | | **Other/Unknown** | | **Total** |
| --- | --- | --- | --- | --- | --- | --- | --- |
|  | **Women** | **Men** | **Women** | **Men** | **Women** | **Men** |  |
|  |  |  |  |  |  |  |  |
| 1998 | 98 | 35 | 45 | 22 | 7 | 4 | 211 |
| 1999 | 103 | 28 | 47 | 17 | 5 | 2 | 202 |
| 2000 | 118 | 41 | 58 | 20 | 5 | 4 | 246 |
| 2001 | 105 | 29 | 54 | 27 | 13 | 3 | 231 |
| 2002 | 130 | 34 | 45 | 30 | 5 | 2 | 246 |
| 2003 | 141 | 42 | 51 | 17 | 3 | 1 | 255 |
| 2004 | 146 | 36 | 41 | 35 | 2 | 0 | 260 |
| 2005 | 153 | 65 | 60 | 26 | 4 | 1 | 309 |
| 2006 | 175 | 70 | 47 | 19 | 10 | 4 | 325 |
| 2007 | 179 | 70 | 63 | 36 | 7 | 2 | 357 |
| 2008 | 236 | 48 | 63 | 22 | 7 | 2 | 378 |
| 2009 | 255 | 78 | 51 | 27 | 6 | 2 | 419 |
| 2010 | 235 | 79 | 76 | 48 | 13 | 4 | 455 |
| 2011 | 312 | 75 | 64 | 24 | 10 | 2 | 487 |
| 2012 | 314 | 97 | 69 | 27 | 11 | 8 | 526 |
| Total | 2700 | 827 | 834 | 397 | 108 | 41 | 4907 |

Data source: National Institute of Cancer Epidemiology and Registration (NICER).
