# Supplementary material for: Overdiagnosis and overtreatment of thyroid cancer: A population-based temporal trend study
Source: PLoS One. 2017 Jun 14;12(6):e0179387. doi: 10.1371/journal.pone.0179387 (PMC5470703; doi:10.1371/journal.pone.0179387)
Supplement: S2 Table — (DOCX) [file pone.0179387.s002.docx]

**Supporting information**

**S2 Table.** Number of thyroidectomy cases by year and sex, Switzerland, 1998-2012.

| **Year** | **Thyroidectomies** | | **Total** |
| --- | --- | --- | --- |
|  | **Women** | **Men** |  |
| 1998 | 108 | 48 | 156 |
| 1999 | 162 | 50 | 212 |
| 2000 | 180 | 53 | 233 |
| 2001 | 213 | 88 | 301 |
| 2002 | 253 | 78 | 331 |
| 2003 | 262 | 80 | 342 |
| 2004 | 290 | 101 | 391 |
| 2005 | 332 | 129 | 461 |
| 2006 | 317 | 121 | 438 |
| 2007 | 390 | 135 | 525 |
| 2008 | 430 | 120 | 550 |
| 2009 | 408 | 140 | 548 |
| 2010 | 398 | 169 | 567 |
| 2011 | 499 | 161 | 660 |
| 2012 | 521 | 183 | 704 |
| Total | 4763 | 1656 | 6419 |

Data source: Swiss Federal Statistical Office (SFSO), Hospital Medical Statistics
